# An association study between *USP34* and polycystic ovary syndrome

**DOI:** 10.1186/s13048-015-0158-y

**Published:** 2015-05-15

**Authors:** Shigang Zhao, Ye Tian, Wei Zhang, Xiuye Xing, Tao Li, Hongbin Liu, Tao Huang, Yunna Ning, Han Zhao, Zi-Jiang Chen

**Affiliations:** Shanghai Key Laboratory for Assisted Reproduction and Reproductive Genetics, Center for Reproductive Medicine, Renji Hospital, School of Medicine, Shanghai Jiaotong University, Shanghai, 200135 China; Center for Reproductive Medicine, Provincial Hospital Affiliated to Shandong University, Jinan, China; National Research Center for Assisted Reproductive Technology and Reproductive Genetics, Jinan, China; The Key laboratory for Reproductive Endocrinology of Ministry of Education, Jinan, China; Shandong Provincial Key Laboratory of Reproductive Medicine, Jinan, 250021 China; Department of joint and bone oncology, Provincial Hospital Affiliated to Shandong University, Jinan, 250021 China

**Keywords:** Polycystic ovary syndrome, USP34, SNPs, Association

## Abstract

**Background:**

Polycystic ovary syndrome (PCOS) is a complex multifactor disorder and genetic factors have been implicated in its pathogenesis. Our previous genome-wide association study (GWAS) had identified allele frequencies in several single nucleotide polymorphisms (SNPs) in gene *USP34* (Ubiquitin-Specific Protease 34) were significantly different between PCOS cases and controls. This study was aimed to replicate the previous results in another independent cohort.

**Methods:**

One thousand two hundred eighteen PCOS cases and 1057 controls were recruited. Genotyping of two SNPs (rs17008097 and rs17008940) in *USP34* gene were performed by TaqMan-MGB probe assay and genotype-phenotype analysis was conducted subsequently.

**Results:**

The differences of allele or genotype frequencies were not significant statistically between PCOS and controls, even after age and BMI adjustment. For clinical and metabolic features (LH, T and HOMA-IR) analysis in PCOS cases, no statistical differences among three genotypes of rs17008097 and rs17008940 were found. However, rs17008940 was shown to be slightly associated with BMI in PCOS cases rather than in controls, even after age adjustment (TC vs CC *P* = 0.006, *OR* = 1.042, 95% CI 1.012–1.073; TT vs CC *P* = 0.037, *OR* = 1.050, 95% CI 1.003–1.100).

**Conclusions:**

*USP34* gene polymorphisms (rs17008097 and rs17008940) may not be associated with PCOS in the Han Chinese women.

**Electronic supplementary material:**

The online version of this article (doi:10.1186/s13048-015-0158-y) contains supplementary material, which is available to authorized users.

## Background

Polycystic ovary syndrome (PCOS) is a kind of reproductive and metabolic disorder characterized by hyper-androgen and insulin resistance, which affects 6–8 % of reproductive-aged women in Caucasian and 5.6 % in Chinese [1–3]. Clinical diagnosis of PCOS is made on the basis of at least two following criteria after excluding other related diseases: oligo- or anovulation, clinical or biochemical hyperandrogenism and polycystic ovaries under ultrasound [4]. The etiology of PCOS is not well understood yet. However, it’s now widely accepted that genetic factors play an indispensable role in the development of PCOS [5] and several candidate genes have been reported recently [6, 7]. We performed the first GWAS for PCOS which followed by replication studies only for SNPs with p value less than 10e-6, and finally identified three susceptibility loci (2p16.3, 2p21 and 9q33.3) [6]. However, other loci with p value around 10e-5 in GWAS, such as SNPs in gene *USP34*, remain intriguing and might also be potential risk factors of PCOS.

The *USP34* gene is located on chromosome 2p15 and encodes a kind of deubiquitinating enzyme, which belongs to ubiquitin-specific protease family. Data obtained from COSMIC database [8] shows that somatic variations of *USP34* are related to ovary tumor. Moreover, USP34 positively regulates Wnt signaling pathway [9], which plays an important role in gender differentiation, folliculogenesis, ovulation and other biological processes in reproduction [10]. The expression patterns of several genes in Wnt pathway are altered in PCOS (such as DKK1, a negative regulator of Wnt pathway) [11–13]. Taken together, it is assumed that *USP34* may also have relationship with PCOS. As an extension of GWAS, here we conducted an independent case-control replication study to evaluate the association between *USP34* and PCOS susceptibility.

## Methods

This study was approved by Institutional Review Board for Reproductive Medicine of Shanghai Jiaotong University and Shandong University. A total of 1218 PCOS cases and 1057 unrelated controls were recruited consecutively from 2009 to 2013 at the Center for Reproductive Medicine, Renji Hospital, School of Medicine, Shanghai Jiaotong University and the Center for Reproductive Medicine, Provincial Hospital Affiliated to Shandong University. Among them, 94 PCOS cases, also born from Northern China, were collected at Renji Hospital from 2012 to 2013. Signed informed consent was obtained from each participant of this study.

PCOS diagnosis was based on the 2003 Rotterdam PCOS consensus criteria and other related diseases (such as congenital adrenal hyperplasia, Cushing’s syndrome, androgen-secreting tumors, thyroid disease and hyperprolactinaemia) were excluded. In detail, PCOS can be diagnosed if at least two of the following three features are met: oligo- or anovulation, clinical and/or biochemical signs of hyperandrogenism and polycystic ovaries. Oligo- or anovulation was referred to menstrual cycles of more than 35 days in length or a history of ≤ 8 menstrual cycles in a year [1]; polycystic ovaries was defined as the presence of at least one ovary >10 ml or at least 12 follicles 2–9 mm in diameter by transvaginal ultrasound [4]. Hyperandrogenism was the presence of hirsutism (Ferriman-Gallwey score ≥ 6) [14] or serum total testosterone ≥ 60 ng/dl [15]. The inclusion criteria for the control group were as follows: normal menstrual cycles, no hyperandrogenism and no polycystic ovaries (PCO) under ultrasound. All individuals who were taking medications such as oral contraceptives and metformin during last 3 months were excluded.

### Biochemical measurements

Serum luteinizing hormone (LH) and testosterone (T) levels of subjects were measured by a chemiluminescent analyser (Beckman Access Health Company, Chaska, Minnesota, USA). The plasma glucose was measured by AU640 automatic biochemistry analyser (Olympus Company, Hamburg, Germany) and insulin was measured by chemiluminescent analyzer. Insulin resistance was calculated as fasting glucose (mmol/L)*fasting insulin (mIU/L)/22.5 using homeostasis model assessment (HOMA-IR).

### SNP selection

SNPs in *USP34* were selected for replication study according to the following criteria: SNPs that exist in Affymetrix Genome-Wide Human SNP Array 6.0 (Affymetrix, Santa Clara, CA, USA); can stand for a block; minor allele frequency (MAF) > 5 % in Chinese Han population; statistically different (*P* < 10e-4) from our previous GWAS (see Additional file 1, Table S1); r^2^ of selected SNPs < 0.8. Ultimately, rs17008097 and rs17008940 were selected to precede further replication study.

### SNP genotyping

Genomic DNA was extracted from whole peripheral blood using QIAamp DNA mini kit (Qiagen, Hilden, Germany). Genotyping of SNPs was carried out by TaqMan-MGB probe assay (Invitrogen Trading, Shanghai, China), probes and primers were shown in Additional file 1, Table S2. Then, 5 % of the samples were randomly selected for direct sequencing to validate the genotyping assays.

### Statistical analysis

Numerical variables of clinical characteristics of PCOS cases and controls were expressed as mean ± SD. Hardy-Weinberg equilibrium (HWE) and linkage disequilibrium (LD) tests were performed by Haploview software. The case-control genetic power was analyzed by Genetic Power Calculator [16]. A sample size > 721 PCOS cases (case: control = 1) would provide 80 % power (*α* = 0.05), assuming higher risk allele frequency (A) of 0.05 and a genotype relative risk (Aa) of 1.5. Frequencies of genotype and allele between PCOS subjects and controls were compared by Pearson Chi-square test, and the p value was adjusted by logistic regression to eliminate the effect of age and body mass index (BMI) using SPSS v.19.0 (SPSS Inc., Chicago, IL, USA). *P* < 0.05 was considered statistically significant.

For genotype-phenotype analysis in PCOS patients, additive model (+/+ vs +/− vs −/−) was selected after comparison; one-way analysis of variance (ANOVA) test was used for phenotype comparison among different genotypes. Linear regression was used for age and BMI adjustment. Conservative Bonferroni test was used for multiple testing corrections.

## Results

As shown in Table 1, the PCOS group was younger than the controls (*P* < 0.001). In addition, PCOS group had higher BMI, LH level and T level than the control group (*P* < 0.001). In PCOS group, 90 patients present with hyperandrogenism and oligo-anovulation (HA + OA, 9.44 %), 10 patients present with hyperandrogenism and polycystic ovaries (HA + PCO, 1.05 %), 620 patients present with oligo-anovulation and polycystic ovaries (HA + PCO, 65.06 %), and 233 patients present with full-phenotype (HA + OA + PCO, 24.45 %).

**Table 1:** Characteristics of PCOS cases and controls

	PCOS	Control	p value
Age (years)	28.55 ± 3.72	31.84 ± 4.74	<0.001
BMI (kg/m^2^)	25.12 ± 4.35	22.78 ± 3.25	<0.001
LH (IU/L)	10.03 ± 5.93	4.76 ± 2.23	<0.001
T (ng/dl)	51.69 ± 21.00	42.35 ± 18.46	<0.001

BMI: body mass index; LH: Luteinizing hormone; T: testosterone

The genotype frequencies of the two polymorphisms in PCOS cases and controls were all in Hardy-Weinberg equilibrium (*P* > 0.05). Genotype and allele frequencies were summarized in Table 2 and no significant differences were observed between PCOS and controls. After age and BMI adjustment with logistic regression, no association was found. The minor allele frequencies (MAF) of the 2 SNPs in four subgroups of PCOS were further analyzed. No significant differences of MAF were observed between each subgroup of PCOS and controls in the present study (see Additional file 1, Table S3). Additionally, there was no statistical difference among three genetic models (additive, dominant and recessive) in genotype analysis (see Additional file 1, Table S4), thus additive model of genotype was selected for subsequent phenotype analysis.

**Table 2:** Genotype and allele frequencies of USP34 in PCOS and controls

			PCOS	Control	*P*	*P*_adjusted_	OR/95 % CI
rs17008097	Genotype	CC	548(45 %)	484(45.8 %)	0.807	0.791	
		GC	531(43.6 %)	447(42.3 %)			
		GG	139(11.4 %)	126(11.9 %)			
	Allele	C	1627(66.8 %)	1415(66.9 %)	0.918	0.791	0.993 (0.878–1.124)
		G	809(33.2 %)	699(33.1 %)			
rs17008940	Genotype	CC	575(47.2 %)	505(47.8 %)	0.948	0.862	
		TC	513(42.1 %)	438(41.4 %)			
		TT	130(10.7 %)	114(10.8 %)			
	Allele	C	1663(68.3 %)	1448(68.5 %)	0.869	0.862	0.990 (0.873–1.122)
		T	773(31.7 %)	666(31.5 %)			

*P* adjusted: adjust the p value by age and BMI; OR: Odd Ratio; CI: Confidence Interval

In genotype-phenotype analysis, clinical and metabolic features were compared among different genotypes in PCOS subjects, rs17008940 was shown to be associated with BMI (*P* = 0.028) (Table 3), however, the association was not significant after Bonferroni correction for multiple testing. The average level of BMI in TC and TT group was higher than that in CC group after age adjustment (TC vs CC *P* = 0.006, *OR* = 1.042, 95 % CI 1.012–1.073; TT vs CC *P* = 0.037, *OR* = 1.050, 95 % CI 1.003–1.100). But in control group, no significant difference was found in BMI among the three genotypes of rs17008940 (*P* = 0.256). Additionally, there were no significant differences in LH, T or HOMA-IR among the PCOS cases carrying different genotypes of the two SNPs, even after age and BMI adjustment (Table 3, Table 4).

**Table 3:** Clinical and metabolic characteristics of PCOS cases in rs17008940 genotype subgroups

Characteristics	CC(*n* = 575)	TC(*n* = 513)	TT(*n* = 130)	*P*	*P*_adjusted_
Age (years)	28.83 ± 3.73	28.39 ± 3.72	28.02 ± 3.66	0.046	−
BMI (kg/m^2^)	24.740 ± 4.29	25.418 ± 4.34	25.517 ± 4.51	0.028	−
LH (IU/L)	9.999 ± 6.08	10.028 ± 5.88	10.177 ± 5.44	0.957	0.500
T (ng/dl)	51.17 ± 20.73	51.74 ± 21.41	53.72 ± 20.61	0.485	0.505
HOMA-IR	2.62 ± 2.62	2.70 ± 2.12	2.79 ± 2.32	0.653	0.399

HOMA-IR: homeostasis model assessment; *P* adjusted: adjust the p value by age and BMI in logistic regression

**Table 4:** Clinical and metabolic characteristics of PCOS cases in rs17008097 genotype subgroups

Characteristics	CC(*n* = 548)	GC(*n* = 531)	GG(*n* = 139)	*P*	*P*_adjusted_
Age(years)	28.82 ± 3.79	28.44 ± 3.64	27.97 ± 3.73	0.044	−
BMI (kg/m^2^)	24.824 ± 4.26	25.287 ± 4.37	25.581 ± 4.51	0.106	−
LH (IU/L)	10.000 ± 6.12	9.963 ± 5.81	10.399 ± 5.61	0.746	0.393
T (ng/dl)	51.446 ± 20.14	51.897 ± 21.97	51.868 ± 20.69	0.940	0.904
HOMA-IR	2.642 ± 2.61	2.676 ± 2.19	2.773 ± 2.24	0.800	0.415

HOMA-IR: homeostasis model assessment; *P* adjusted: adjust the p value by age and BMI in logistic regression

## Discussion

As a powerful technique, GWAS shed new light on genetic study for complex diseases. GWAS data is obtained from computing and statistical analyses following SNP chips detection, so the results are bioinformatics rather than biological. However, GWAS itself owns some limitations and it is necessary to be validated through further replication studies. In general, p value of 5*10e-8 was used as significant level for random variations in case-control GWAS with a power of 0.8 [17, 18]. In our previous GWAS, only SNPs with p value < 10e-6 were replicated to confirm the first step results [6]. However, some SNPs with p value around 10e-5 were disputable. Recently we found two novel susceptibility genes *YAP1* and *LPP* for PCOS from these SNPs [7, 19]. So we selected two SNPs with p value around 10e-5 in *USP34* to validate whether *USP34* was associated with PCOS. No association was replicated in the present study. Therefore, SNPs with p value around 10e-5 in GWAS were controvertible and demanded for independent and large cohort of samples for verification.

Although rs17008940 was not shown to be related to PCOS, it might confer slight risk to the elevated BMI in PCOS. However, this slightly association possibly results from a selection bias derived from the patients and controls being recruited in an infertility clinical center and not from the general population. Hence, the results need further validation. Higher BMI was one of the important characteristics in PCOS and over 50 % PCOS women were overweight or obesity [20, 21]. Consistent with our results, previous studies also showed *FTO* and *MC4R* were associated with increased BMI in PCOS subjects rather than PCOS itself [22]. Abundant evidence have linked Wnt signals to the regulation of adipogenesis [23, 24] and body fat distribution [25]. For example, Christodoulides et al. reported that mutation C256Y in WNT10B was associated with overweight or obesity because the mutation was unable to activate canonical Wnt pathway [26]; and Choi et al. found that indirubin-3′-oxime (I3O), also an activator of the Wnt signaling like USP34, inhibited the development of obesity in high-fat diet fed mice [27]. Moreover, besides acting as an activator of Wnt pathway, USP34 was also found to play a role in NFκB signal regulation in T lymphocytes [28] and DNA damage response control as it was the downstream of ATM/ATR checkpoint kinase [29, 30]. USP34 may indirectly participate in the pathophysiology of PCOS by elevating BMI, but further studies were still needed to evaluate the function of USP34 in the BMI increase among PCOS women.

Some limitations of the present replication study should be mentioned. First, the sample size of this replication study was relatively small (rs17008940, *OR* = 1.010; rs17008097, *OR* = 1.007) and this replication study maybe not sufficient to detect the potential association between *USP34* gene and PCOS. Second, only 2 SNPs were chosen which may cause incomplete coverage of the gene variations. Third, the recruited subjects were all Han Chinese women and the result could not represent other population.

## Conclusions

In conclusion, the present study found that polymorphisms of *USP34* gene may not be associated with PCOS women among Han Chinese population. SNPs with p value around 10e-4 ~ 10e-6 in GWAS were disputable and requiring replication studies for validation. Large well-designed and population-based studies are warranted to confirm our findings.

## Additional file

Additional file 1: Table S1. GWAS database of USP34. **Table S2.** Probes and primers of the three SNPs. **Table S3.** Allele frequencies comparison of USP34 in four subgroups of PCOS and controls. **Table S4.** Genotype frequencies comparison in PCOS and controls using different genetic models.
