# Supplementary material for: An association study between USP34 and polycystic ovary syndrome
Source: J Ovarian Res. 2015 May 15;8:30. doi: 10.1186/s13048-015-0158-y (PMC4435648; doi:10.1186/s13048-015-0158-y)
Supplement: Additional file 1: Table S1. — GWAS database of USP34. Table S2. Probes and primers of the three SNPs. Table S3. Allele frequencies comparison of USP34 in four subgroups of PCOS and controls. Table S4. Genotype frequencies comparison in PCOS and controls using different genetic models. [file 13048_2015_158_MOESM1_ESM.pdf]

Additional file 1

Table S1: GWAS database of *USP34*

SNP	Allele	F_A	F_U	CHISQ	P
rs777587	G/A	0.3014	0.2732	3.086	0.07898
rs4671398	A/G	0.2545	0.2392	0.9981	0.3178
rs11889205	A/G	0.01448	0.01134	0.625	0.4292
rs2290324	C/A	0.3971	0.3719	2.139	0.1436
rs13388389	C/A	0.01379	0.01134	0.3903	0.5321
rs7591772	G/C	0.3029	0.2716	3.742	0.05308
rs17007356	A/C	0.3012	0.3611	12.79	**3.49E-04**
rs2167565	C/T	0.2462	0.2333	0.7331	0.3919
rs10496091	T/C	0.01655	0.0119	1.245	0.2645
rs2167566	G/T	0.3953	0.3688	2.367	0.1239
**rs17008097**	G/C	0.2983	0.3576	12.47	**4.14E-04**
rs12622458	C/A	0.399	0.3697	2.881	0.08962
rs2600672	A/G	0.3946	0.3622	3.541	0.05989
rs2694619	C/T	0.2993	0.2775	1.836	0.1755
rs2694618	G/A	0.3022	0.2766	2.533	0.1115
rs17482440	C/T	0.3024	0.2768	2.521	0.1123
rs778157	C/G	0.3968	0.3683	2.73	0.09851
rs778160	G/A	0.2764	0.2517	2.458	0.117
rs778143	A/C	0.3947	0.3608	3.894	0.04845
rs1584301	C/T	0.3057	0.373	16	**6.34E-05**
rs2694643	A/G	0.3942	0.3617	3.579	0.05852
rs17008755	C/T	0.2967	0.3652	16.68	**4.43E-05**
rs10208769	A/T	0.3022	0.2713	3.727	0.05354
**rs17008940**	T/C	0.2766	0.343	16.33	**5.33E-05**
rs10496093	T/C	0.281	0.3465	15.52	**8.15E-05**
rs812925	C/G	0.203	0.186	1.475	0.2246
rs778139	A/G	0.2167	0.197	1.863	0.1723

P: p value

Table S2: Probes and primers of the three SNPs

SNPs	Probes	Primers
rs17008097	FAM-TAAGTAGTTTATTTCAGGACATC-MGB	F-GCTCCTGGCTTAAAGATTGTTTTT
	VIC-TAAGTAGTTTATTTGAGGACATC-MGB	R-TTCATTCATCCTGTAGAGTTCTTTCG
rs17008940	FAM-ACGGACTAACGTTACTGA-MGB	F-AGGGCAGCCGTTTGTAAAGA
	VIC-ACGGACTAACATTACTGAA-MGB	R-CCCAGGAAATGCTATAAACATGTTC

F: forward; R: reverse

Table S3 Allele frequencies comparison of *USP34* in four subgroups of PCOS and controls.

		Control	HA+OA+PCO (n=233)	HA+OA (n=90)	HA+ PCO (n=10)	OA+PCO (n=620)
rs17008097	MAF	0.331	0.325	0.382	0.35	0.328
	OR	-	1.03	0.80	0.92	1.01
	(95%CI)		(0.83-1.27)	(0.58-1.10)	(0.36-2.31)	(0.87-1.17)
rs17008940	MAF	0.315	0.306	0.365	0.35	0.339
	OR	-	1.05	0.80	0.85	0.90
	(95%CI)		(0.84-1.30)	(0.58-1.10)	(0.34-2.15)	(0.78-1.04)

HA: hyperandrogenism; OA: oligo-anovulation; PCO: polycystic ovaries; OR: Odd Ratio; CI: Confidence Interval.

Table S4: Genotype frequencies comparison in PCOS and controls using different genetic models

SNP	Genotype	PCOS	Control	P_{add}	P_{dom}	P_{rec}
rs17008097	CC/GC/GG	548/531/139	484/447/126	0.807	0.706	0.703
rs17008940	CC/TC/TT	575/513/130	505/438/114	0.948	0.931	0.787

P_{add}: statistic difference of additive model (the three genotypes, +/+ vs. +/- vs. -/-); P_{dom}: dominant model (homozygote of major allele frequency + heterozygote compared with homozygote of minor allele frequency, +/+ plus +/- vs. -/-) of genotype; P_{rec}: recessive model (homozygote of minor allele frequency compared with heterozygote+ homozygote of minor allele frequency, +/+ vs. +/- plus -/-) of genotype
